# Rehabilitation Before and After Autologous Haematopoietic Stem Cell Transplantation (AHSCT) for Patients With Multiple Sclerosis (MS): Consensus Guidelines and Recommendations for Best Clinical Practice on Behalf of the Autoimmune Diseases Working Party, Nurses Group, and Patient Advocacy Committee of the European Society for Blood and Marrow Transplantation (EBMT)

**DOI:** 10.3389/fneur.2020.556141

**Published:** 2020-12-11

**Authors:** Fiona Roberts, Helen Hobbs, Helen Jessop, Cristina Bozzolini, Joachim Burman, Raffaella Greco, Azza Ismail, Majid Kazmi, Kirill Kirgizov, Gianluigi Mancardi, Susan Mawson, Paolo A. Muraro, Mathieu Puyade, Riccardo Saccardi, Barbara Withers, Bregje Verhoeven, Basil Sharrack, John A. Snowden

**Affiliations:** ^1^Hobbs Rehabilitation, Winchester, United Kingdom; ^2^Sheffield Teaching Hospitals NHS Foundation Trust, Sheffield, United Kingdom; ^3^Careggi University Hospital, Florence, Italy; ^4^Department of Neuroscience, Uppsala University, Uppsala, Sweden; ^5^Hematology and Bone Marrow Transplantation Unit, Istituto di Ricovero e Cura a Carattere Scientifico (IRCCS), San Raffaele Scientific Hospital, Milan, Italy; ^6^Kings Health Partners, Department of Haematology, Guys Hospital, London, United Kingdom; ^7^Institute of Paediatric Oncology and Haematology, N.N. Blokhin National Medical Research Center of Oncology, Moscow, Russia; ^8^Department of Neuroscience, University of Genova and Clinical Scientific Institutes Maugeri, Genoa, Italy; ^9^School of Health and Related Research, University of Sheffield, Sheffield, United Kingdom; ^10^Department of Brain Sciences, Imperial College London, London, United Kingdom; ^11^CHU de Poitiers, Service de Médecine Interne et Maladies Infectieuses, Poitiers, France; ^12^CHU de Poitiers, CIC-1402, Poitiers, France; ^13^Department of Haematology and Blood Stem Cell Transplantation, St Vincent's Health Network Sydney and Senior Lecturer, St Vincent's Clinical School, University of New South Wales Medicine, Sydney, NSW, Australia; ^14^Patient Advocacy Committee, EBMT Executive Office, Barcelona, Spain

**Keywords:** autoimmune diseases, autologous haematopoietic stem cell transplantation, neurological diseases, multiple sclerosis, rehabilitation, physical therapy, exercise

## Abstract

Autologous haematopoietic stem cell transplantation (AHSCT) is increasingly used to treat people with multiple sclerosis (MS). Supported by an evolving evidence base, AHSCT can suppress active inflammation in the central nervous system and induce long-term changes in immune cell populations, thereby stabilizing, and, in some cases, reversing disability in carefully selected MS patients. However, AHSCT is an intensive chemotherapy-based procedure associated with intrinsic risks, including profound cytopenia, infection, and organ toxicity, accompanied by an on-going degree of immuno-compromise and general deconditioning, which can be associated with a transient increase in functional impairment in the early stages after transplantation. Although international guidelines and recommendations have been published for clinical and technical aspects of AHSCT in MS, there has been no detailed appraisal of the rehabilitation needed following treatment nor any specific guidelines as to how this is best delivered by hospital and community-based therapists and wider multidisciplinary teams in order to maximize functional recovery and quality of life. These expert consensus guidelines aim to address this unmet need by summarizing the evidence-base for AHSCT in MS and providing recommendations for current rehabilitation practice along with identifying areas for future research and development.

## Introduction

### Multiple Sclerosis

Multiple Sclerosis (MS) is an acquired chronic immune-mediated inflammatory disease of the central nervous system (CNS) and is the commonest cause of non-traumatic disability in adults of working age (1, 2).

Approximately 85% of people with MS present with a relapsing remitting course (RRMS) characterized by distinct episodes of new or worsening neurological dysfunction (relapses) followed by complete or partial recovery (remission) (3). In the majority of cases, patients transition into a secondary progressive phase (SPMS) 10–25 years after the disease onset, accumulating progressive disability (4). A minority of patients (10–15%) present with a progressive disease course from the onset of disease, described as primary progressive MS (PPMS) (4).

Clinical presentation varies dependent on the site of lesions within the CNS. Clinical features may include abnormalities of muscle strength, sensation, balance, coordination, vision, cognition, speech, swallowing, bladder, bowel, and sexual function as well as tremor, pain, fatigue, heat sensitivity, and changes in mood and personality (5). MS can be highly disabling with considerable personal, social, and economic consequences (6, 7).

## Medical Management of Multiple Sclerosis

Currently, there is no known cure for MS. Medical treatment aims to modify the course of the disease process and control symptoms as they develop (8). In RRMS, treatment is aimed at reducing permanent damage to the CNS by decreasing inflammation and preventing relapses (8). There are now 15 licensed disease-modifying therapies (DMTs), which aim to suppress inflammation and prevent the progressive phase of disease (9). Steroids are frequently used to manage relapses in the acute setting, but have no beneficial long-term effect. The treatment options for patients with SPMS and PPMS are very limited.

DMTs have various levels of efficacy but many patients respond poorly and continue to accumulate disability (10). There is also a risk of immuno-compromise and other toxicities. In recent years increasing numbers of patients with MS have been treated with autologous HSCT (11–13). Specific EBMT guidelines have been published to assist with patient selection, advice about transplant protocols, and supportive care (14).

## AHSCT and Multiple Sclerosis

In recent years an increasing evidence base and published professional guidelines have supported more widespread use of AHSCT as a treatment option in patients with highly active RRMS. MS is now the fastest growing indication for AHSCT across Europe (15).

AHSCT provides a “one-off” treatment as opposed to DMTs, which generally require on-going administration (7, 16). AHSCT, however is an intensive chemotherapy-based procedure that can result in deconditioning and which may add further to functional impairment, at least temporarily (17). AHSCT and its associated short- and long-term risks require counseling, detailed workup and an admission to specialized HSCT facilities (18). The provision of a coordinated care plan derived jointly by transplant hematologists, MS neurologists, and other allied healthcare professionals is essential throughout the procedure (19).

Although AHSCT is not thought to be directly associated with repair or regeneration of damaged myelin or nerve fibers, previous studies have demonstrated reversal of disability in some patients following treatment which could be explained by the prolonged suppression of inflammation and physiological CNS repair (19). Secondary benefits of AHSCT include reduced fatigue and improved energy reserves in contrast to profound fatigue, which is commonly associated with active inflammation (20). At present there are no studies to confirm whether neuro-rehabilitation may promote recovery following AHSCT (21), but this may be a reasonable extrapolation from standard neuro-rehabilitation practice. AHSCT has the capacity to halt the inflammatory process for an extended period of time, which may allow the CNS to acquire the capacity to repair and re-organize the damaged areas. Neuro-rehabilitation can contribute to this.

## Neuro-Rehabilitation in MS

Outcomes for individuals with MS could be maximized through rehabilitation; both by optimizing a patient's physical fitness and by guiding and stimulating neuroplasticity. In MS, evidence suggests that neuroplasticity can play a role in limiting the clinical impact of damage (22). Task orientated interventions can result in the reorganizing or restoration of altered patterns of brain activity and may induce clinically meaningful re-myelination and plasticity changes (23). In MS, maladaptive plasticity can occur in the CNS following neuronal injury (23), highlighting the need for specialist therapy teams to guide rehabilitation intervention.

With AHSCT, there is a rapid and prolonged suppression of CNS inflammation associated with a sustained interval free of immunosuppressive treatments; therefore, the early administration of a neuro-rehabilitation program represents a unique opportunity to maximize its effect, possibly in association with axonal repair.

Similar to the medical management for MS, rehabilitation intervention is determined by the symptoms and clinical presentation for each patient and therefore a full clinical assessment is essential with the rehabilitation being goal directed and person centered (24, 25). This should be multi-disciplinary with involvement from neurologists, specialist nurses, rehabilitation specialists, physiotherapists, occupational therapists, speech and language therapists, and neuro-psychologists. Where possible, the rehabilitation must involve self-management and where possible family and carers to ensure a 24 h approach.

## The Unmet Need: Guidelines and Recommendations for Rehabilitation of Patients Undergoing AHSCT for MS

Currently, there is no established rehabilitation pathway for patients with MS either before or after AHSCT. Although pre-habilitation and rehabilitation have been explored in malignant hematological diseases where AHSCT is routinely used (26), there is very limited evidence in relation to AHSCT in MS. In addition, there is no consensus as to how rehabilitation should be delivered and provision will vary from country to country. Now that there is a significant demand across health services in many countries for AHSCT in patients with MS (14), addressing this unmet need has become an urgent matter. Therapists need clear guidelines to advise when they should start rehabilitation, to what intensity, how to address the potential complications associated with the AHSCT procedure and how to tailor rehabilitation programs to suit each individual's symptoms and goals. Rehabilitation plans need to factor in patients' initial presentation and their positive and negative response to AHSCT particularly if they become systemically unwell and deconditioned during the treatment period.

## Aims and Process for Guidelines and Recommendations

The following guidelines and recommendations have been established by consensus to support therapists with the rehabilitation of patients with MS undergoing AHSCT with the goal of optimizing management and overall outcome.

### Methodology

Following approval by the processes of the EBMT Autoimmune Diseases Working Party (ADWP), an authorship group was convened from clinicians from relevant professional groups active in or associated with the ADWP and Nurses Group (NG) of the European Society for Blood and Marrow Transplantation (EBMT) with experience in AHSCT for neurological ADs as well as patient consumer representation via the EBMT Patient Advocacy Committee. As such, this group included therapists, hematologists, neurologists, specialist nurses, and patient representation, with access to patients with MS who had undergone AHSCT. An important point is that several areas of specialism have collaborated to share expertise in respective areas.

In the absence of specific studies or guidelines for rehabilitation before or after HSCT for MS, based on knowledge within the authorship group and literature searching, the aim was to produce an expert consensus as a starting point. A systematic review was considered to be premature and therefore was not undertaken. Whilst the structure of the guideline and recommendations broadly considered the principles of the AGREE process (27), this was not a systematic review nor did it address specific health question or adhere to the AGREE or similar process. The output was therefore in line with other EBMT guidelines and recommendations, including a recent “White Paper Report” (26) developed to guide rehabilitation in patients who have or are due to receive AHSCT which focused on allogeneic HSCT and graft-versus-host disease.

The target readership primarily includes specialist therapists directly involved in planning rehabilitation for patients with MS undergoing AHSCT as well as clinical teams and their members involved in the planning and delivery of AHSCT. As such the guidelines are written in a technical style for the target readership of health professionals, and, although they are not intended as a primary resource for patient information, they should be made accessible to them via their health professionals. These guidelines cover adults, young people, and children. They are intended to be holistic aiming to address physical rehabilitation as well as cognitive, communication, and psychosocial factors. Specific evidence in the area of rehabilitation in AHSCT for MS is very limited, and therefore all recommendations are consensus and based on agreed best practice within the group, whilst the need for future systematic research in specific areas is recognized.

The aim of these guidelines and recommendations is to suggest a pathway for rehabilitation, recognizing the challenges that patients face at each stage. A further aim was to generate questions for future research in this field. We have divided the rehabilitation process into four phases:

– **Phase 1**: Assessment and *pre-habilitation 4 weeks before starting treatment*,
– **Phase 2**: *Acute rehabilitation*,
– **Phase 3**: *Sub-acute rehabilitation, and*
– **Phase 4**: *Community rehabilitation including vocational rehabilitation*.

Each phase of the pathway has a different emphasis but each treatment plan must still be tailored to the individual and will change depending on the stage of a patient's rehabilitation. These guidelines therefore outline the assessment process and propose outcome measures and standardized assessment tools as well as guidance on therapeutic interventions.

## Selection Criteria for AHSCT for Patients With MS

Patients with MS being considered or offered AHSCT should meet the criteria suggested in current EBMT guidelines and recommendations and further updates, and treatment should be approved by an appropriately constituted multi-disciplinary team (MDT) (14).

## The Multidisciplinary Team (MDT)

In line with EBMT recommendations, care should be provided with a co-ordinated multidisciplinary approach by a team responsible for initial assessment of suitability for AHSCT, and an extended team, who facilitate referral, assessment, and delivery of rehabilitation throughout the four phases of the pathway.

The MDT membership should include:

- Neurologist(s) with an interest in MS
- Hematologist(s) with an interest in HSCT
- Specialist MS nurse(s)
- Specialist HSCT nurse(s)
- Rehabilitation specialist(s)
- Specialist therapists (acute, sub-acute and community based) including physiotherapists, occupational therapists, speech and language therapists
- Neuropsychologists and counselors
- Dieticians
- Pain management specialists
- Infectious disease specialists
- Other specialist nurses including continence and tissue viability nurses, late effects nurses
- GPs and district nursing teams.

## AHSCT – The Process

For detailed general and MS specific literature on AHSCT, please refer to EBMT guidelines for AHSCT in MS and neurological diseases (14, 17, 28, 29) and recent reviews, which cover the transplant process itself and its us in MS.

**Figure 1:**
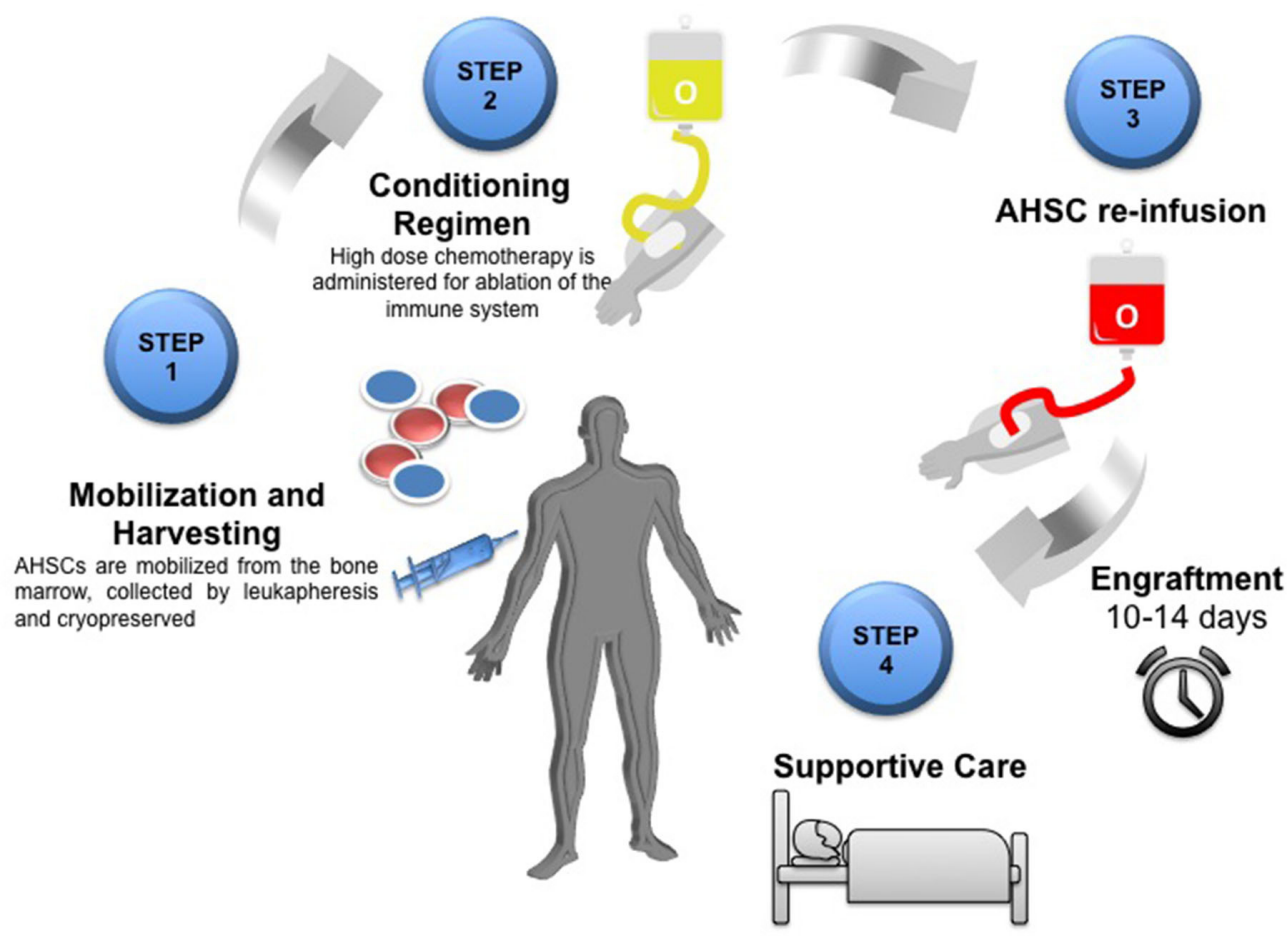
Schematic overview of the AHSCT process *Step one:* Mobilization of AHSCs from the bone marrow by leukapheresis and cryopreserved until required. *Step two:* Conditioning regimen using high dose chemotherapy for ablation of the immune system. *Step 3*: Re-infusion of autologous haematopoeitic stem cells (AHSC). Engraftment of bone marrow takes 10–14 days. *Step 4:* Post-transplant care and supportive therapy.

The process is summarized in Figure 1 and as follows:

1. *Pre-transplant workup, including wash out of DMTs* Patients should be reviewed by both MS neurologists and transplant Hematologists who are experienced in using AHSCT in this context. Patients are initially assessed and provided with extensive counseling to ensure that they are familiar with the benefits and risks of AHSCT (18, 30). Both short- and long-term risks including the effect on reproductive function and other late effects need to be considered (31). The individual is usually taken off their DMT at variable time points prior to the stem cell mobilization, dependent on the specifice DMT (14).
2. *Peripheral blood stem cell [PBSC] mobilization and leukapheresis* In the weeks to months prior to transplant, the patient undergoes a stem cell mobilization procedure, which allows stem cells to be procured for later transplantation (29).
3. *Conditioning regimen* Depending on the protocol, the patient is generally hospitalized from the start of the intensive cytotoxic “conditioning” regimen, which usually includes a combination of high-dose chemotherapy and antibody-based therapy [such as anti-thymocyte globulin (ATG)] which results in ablation of haematopoietic and immune cells throughout the body (17). Usually a central venous line is inserted to facilitate administration of cytotoxic drugs and transfusions. The line may occasionally remain *in situ* beyond the AHSCT procedure and its presence may need consideration when planning rehabilitation.
4. *The “transplant”* The cryopreserved stem cells are thawed and infused through the central venous line with close monitoring of the patient in case of reaction toe the infusion.
5. *Post-transplant care* Following stem cell infusion, the patient remains in hospital for a period of close monitoring, supportive care for side effects of treatment, and isolation from potential sources of infection while awaiting engraftment of the haematopoietic system, usually defined as the recovery of a neutrophil count of 10^9^/L for three days (17), which in most cases occurs 10–14 days following the infusion of the stem cells.

Patients are usually discharged from hospital within 4 weeks, although this depends on their baseline condition and tolerance of AHSCT. Potential early side effects include fever, infection, and sepsis in association with leukopenia, other cytopenias (anemia and thrombocytopenia) requiring blood product transfusions, and a range of other organ toxicities (alopecia, mucositis, diarrhea, vomiting, renal, and fluid balance issues and serum sickness). Worsening of existing neurological symptoms can occur often in the context of fever, drug reactions, and infection (17). Neurological deterioration in response to fever is known as the *Uhthoff* phenomenon, and, whilst the effect on patients during AHSCT is usually short lived and reversible, some evidence suggests the potential for prolonged deleterious effect on neurological function (32). Thus, a proactive approach is appropriate during AHSCT including prompt administration of antibiotics and steroid treatment to prevent or minimize the duration of fever (32).

Secondary complications include deconditioning, reduced caloric intake due to nausea and mucositis and limited activity in the immediate post-transplant period (32). Prolonged bed rest and isolation during AHSCT may also lead to pressure sores, thromboembolic risks, changes in spasticity and posture, acquired urinary or bowel dysfunction, bone loss, vitamin D deficiency, low mood, and fatigue (17).

Long-term effects include risks of infertility (31), autoimmune dysfunction including thyroid abnormalities and autoimmune cytopenias, endocrine, and other organ impairment and secondary malignancies (28).

## Rehabilitation Within the AHSCT Pathway

Utilizing a biopsychosocial model, rehabilitation aims to optimize a patients' health, functional independence and well-being, comprising a comprehensive programme covering the physical, cognitive, psychological, and social aspects of their care. Previous recommendations have advocated the need for physical therapy before and after HSCT to promote recovery of functional capacity and improve quality of life (26). These were aimed at a wide range of hematological conditions and did not include the special considerations for neurological conditions including MS.

The paucity of evidence in the field of rehabilitation means that it is not possible to make firm recommendations. However, a summary of the basis for AHSCT alongside consensus guidance is presented to support therapists and clinicians caring for these patients to optimize collaborative care and identify areas for future development.

**Figure 2:**
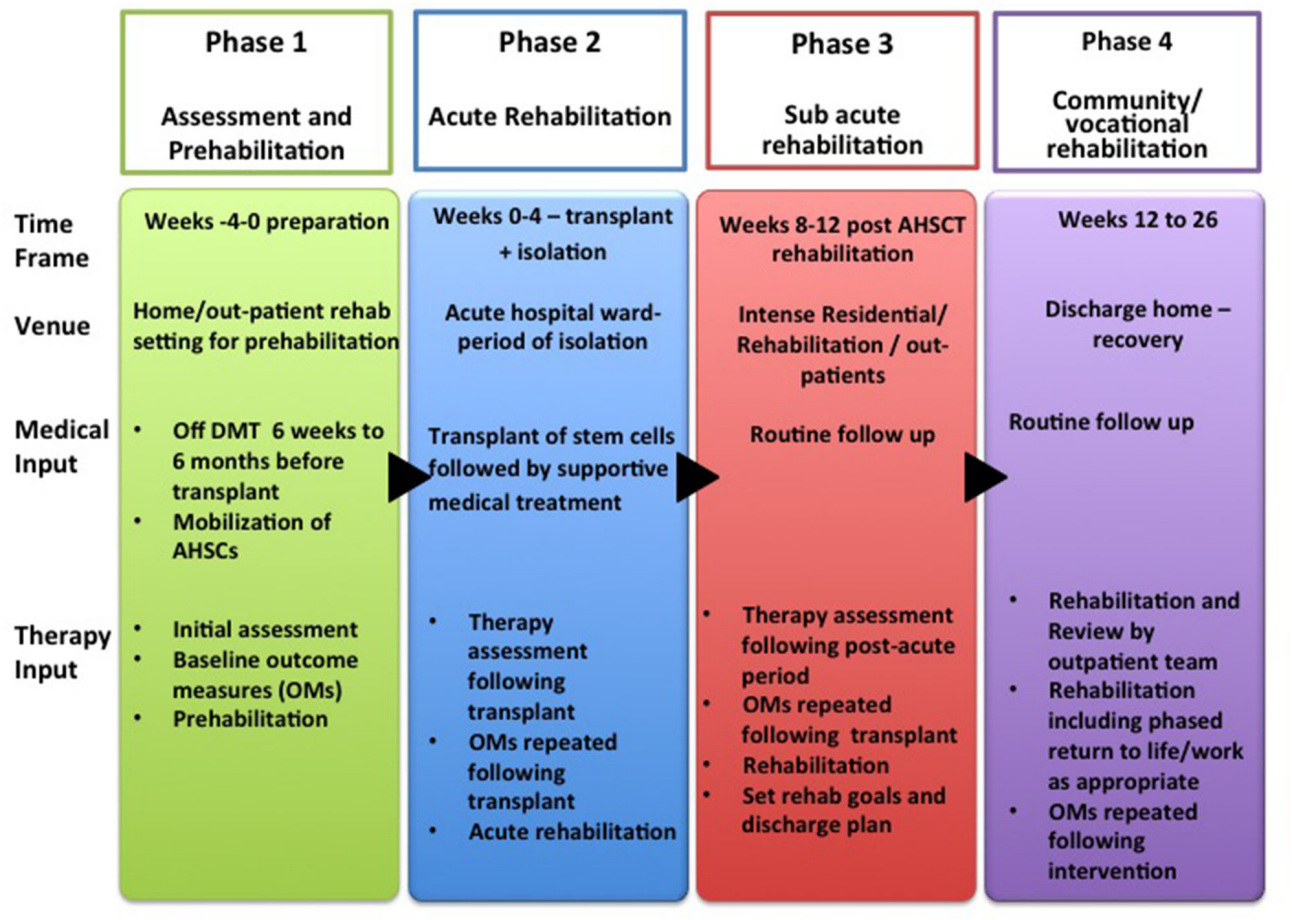
Rehabilitation Pathway for MS patients undergoing AHSCT (*AHSCT, Autologous Haematopoietic Stem cell Transplantation*; *OMs, Outcome Measures; Rehab, Rehabilitation*).

In these guidelines, rehabilitation for patients with AHSCT is delivered over 4 distinct phases during the pathway as detailed above and in Figure 2. Whilst traditionally rehabilitative interventions are delivered during and after treatment, these guidelines recommend an initial “pre-habilitation” phase in line with current evidence in the field of cancer rehabilitation. Pre-habilitation would occur before “*beginning of acute treatment and would include physical and psychological assessments that establish a baseline functional level, identifies impairments, and provides targeted interventions that improve a patient's health to reduce the incidence and the severity of current and future impairments”* (33). We suggest that pre-habilitation is a potential solution to address the needs of these MS patient to improve their outcomes.

In general terms, patients who receive enhanced therapy whilst in acute care go home sooner (34). Rehabilitation in sub-acute and long-term follow up phases may further improve functional outcomes and reduce the burden of long-term care costs associated with dependency but further research is needed to determine the benefits (21, 34).

## Rehabilitation Model

Effective rehabilitation is holistic, multi-disciplinary, goal directed, patient centered, and, where possible, evidence based, as recommended in the various international rehabilitation guidelines for patients with MS including the NICE guidelines (35), the Cochrane review of 2019 (25) and European Multiple Sclerosis Platform Rehabilitation Recommendations (36) to name a few. It is essential that the rehabilitation is adapted to patients' changing needs as they progress.

A series of reliable and validated outcome measures (OMs) should be recorded at key points throughout the patient's journey to monitor any change in key domains within the biopsychosocial model of care as outlined by the World Health Organization's International Classification of Functioning, Disability and Health (37).

### The Assessment

Patients should undergo a detailed assessment (25, 35) at the start of each phase of rehabilitation not only looking at a patient physically but also their cognition, psychology, speech, function, and the utilization of standardized assessments tools and outcome measures where possible is encouraged. This will be patient specific depending on their presentation and will be phase specific.

### Standardized Assessment Tools and Outcome Measures (OMs)

There are no tools or OMs specifically available for use for patients with MS receiving AHSCT and future research into this area is needed. Whilst these guidelines suggest a spectrum of tools and OMs, these are not exhaustive and must be used based on a patient's presentation and their phase of rehabilitation. For example, a patient may not be able to mobilize acutely in the initial days following their transplant, and a 10 m walk test will only be appropriate when they progress and start to mobilize.

To assess someone's cognitive function, there are a number of standardized assessment tools. For example, the Minimal Assessment of Cognitive Function in MS (MACFIMS), and the Montreal Cognitive Assessment (MoCA) have been shown to be a valuable screening tool for MS patients (38, 39). The Brief International Cognitive Assessment for Multiple Sclerosis (BICAMS) was specifically designed with MS patients in mind (40). Further examples of cognitive assessments include the Loewenstein Occupational Therapy Cognitive Assessment (LOCTA) (41), the Rivermead Memory Team (42), the Behavior Assessment of the Dysexecutive Syndrome (43) and the TEA—Test of Everyday Attention which are not specific to MS (44).

For some patients, an assessment of their cognition through function may be more beneficial. For speech therapy, there are assessments for dysarthria for example the Frenchay Dysarthria Assessment (FDA-2) (45) and for language, the Mount Wilga High Level Language Assessment but neither are exclusive to the MS population.

Much like the assessment tools, a series of reliable and validated outcome measures (OMs) should be recorded at key points throughout the transplantation treatment to monitor any change in key domains within the biopsychosocial model of care as outlined by the World Health Organization's International Classification of Functioning, Disability and Health (37). In accordance with our recommendations, OMs should be used at the start and end of each phase and when there is significant change.

The National Multiple Sclerosis Society suggested a number of measures (46) but Cohen et al. described a clinical decision making process when selecting outcome measures (47). However, to date, no research has been conducted to recommend specific OMs for the MS population undergoing AHSCT. Therefore, our recommendations cover those OM with evidence base specifically in patients with MS. The Academy of Physical Therapy in 2012 (48–50) suggested the following OMs in patients with MS:

– 10 m walk test
– Berg balance
– Modified fatigue impact scale
– Rivermead Mobility index
– MS QOL 54 measure
– Dynamic gait index
– Activities of daily living (ADL)
– Nine hole peg test.

## Rehabilitation During Phase 1: Assessment and Pre-Habilitation−4 Weeks to Start of Treatment

### Assessment

Prior to the transplant, it is recommended that baseline level of impairment function and participation is ascertained through a formal MDT assessment process. This should include a full medical and physical assessment, including a medication and social history A physical assessment should include joint range of movement, muscle strength, length and tone, balance, sensation, cerebellar signs, proprioception, movement analysis, and endurance including respiratory function. The patient's cognition, swallowing, communication, nutritional status, functional independence, and endurance should also be assessed including determining how they transfer, mobilize, and complete personal activities of daily living including washing, dressing, feeding as well as their ability to do domestic tasks. A range of standardized outcome measures normally form part of the full assessment (see above).

### Risk Assessment

It is important that any risk factors for potential deterioration are identified. These may include fatigue, existing muscle weakness or spasticity, reduced respiratory function, poor cough, contractures, pressure sores, postural changes, urinary tract infections and other infections, constipation, deconditioning, compromised nutrition, and pain (26). As well as MS, there may be co-morbidities that heighten the risk of these potential complications.

### Advice and Information

In Phase 1, the key focus is optimizing the patients' physical, social, and emotional functioning and well-being prior to them undergoing AHSCT. Patients should be given individualized advice and education to help them self-manage their rehabilitation and prevent risks occurring. Advice leaflets provide guidance on exercise, nutrition, and any necessary lifestyle changes supporting a self-care and self-management paradigm. If appropriate, carers and family members should also be provided with advice and education to support the individual. Specialist consultation may be necessary/beneficial in some cases.

### The Rehabilitation— “Pre-habilitation”

During phase 1, “Pre-habilitation,” can be provided as part of an intensive community rehabilitation package or in an outpatient setting. Residential rehabilitation may be required based on a patient's presentation. Pre-habilitation during this phase will be patient centered based on a full assessment.

The aims of this stage are not only to enhance neuromuscular systems and respiratory function but also to reduce the risk of secondary complications. The following should be considered, as appropriate:

– Breathing exercises and the use of respiratory adjuncts to optimize respiratory function (51).
– Cardiovascular exercise. The evidence suggests that adults with MS should engage in at least 30 min of moderate intensity aerobic activity twice a week as well as strength training of major muscle groups biweekly to optimize fitness (52). High impact weight bearing exercises such as hopping, jumping, combination exercises should also be considered if appropriate as these have a positive impact on bone mass density (53). Pre-transplant fitness can have a positive influence on recovery (34). Access to appropriate gym equipment such as a cross-trainer or static bike to enhance physical fitness may be required. Care should be taken to ensure that the level of exercise prescribed reflects the patient's clinical presentation, over-exertion could have a negative impact. The extent of cardiovascular exercise and the patient's ability to use equipment will depend on their presentation and pre-morbid level of function (54). Stairs or mobility may provide adequate cardiovascular exercise at this stage.
– Strengthening and stretching programmes (55)
– Spasticity management (35)
– The use of neuro-technologies (56)
– Cognitive rehabilitation (35, 57, 58),
– Nutrition (35),
– Relaxation (36),
– Fatigue management (35),
– Pain management (35)
– Providing strategies to accomplish transfers and ADL (34) and
– The provision of equipment including aids and orthotics as appropriate.

All activities should be underpinned by a self-management approach, where possible.

## Rehabilitation During Phase 2: Acute Rehabilitation

Rehabilitation during this phase will be goal directed and patient centered helping prevent secondary problems through gentle mobilization. As such it aims to prevent cachexia and optimize respiratory function. At this stage, the provision of appropriate levels of exercise will be influenced by platelet count and fatigue. In most cases platelet count will have recovered by day +15 post-transplant although secondary immune thrombocytopenia may occur in 5–7% of patients later on. Mental health and well-being should be monitored throughout this phase.

The main consideration for rehabilitation at phase 2 is the individual's immunity. The immune system begins to recover around 2 weeks after infusion of the blood stem cells, during which patients are likely to remain in hospital for close monitoring and continuation of supportive treatment such as antibiotics. Even with neutrophil recovery, the immune system remains suppressed for several months. Any infections may adversely affect patients physically and cognitively (17). Infection control measures should be observed. As this patient group is at risk of urinary dysfunction, there may be a risk of urosepsis and rarely haemorrhagic cystitis and patients may require urinary catheterisation (59).

### Contraindications and Precautions to Rehabilitation

Following AHSCT, patients invariably have cytopenias increasing the risk of infection and bleeding. At this time, physical function does need to be monitored so it is essential that skilled therapists are involved in guiding rehabilitation.

Thrombocytopenia is sometimes considered a contraindication to exercise and therefore platelet count should be monitored and exercise prescribed as appropriate. A previous consensus publication (26) has proposed the following scale for general HSCT patients.

– <20 × 10^9^/L contraindication to exercise
– 20–30 × 10^9^/L—gentle non-resistant exercises
– 30–50 × 10^9^/L—minimal resistance (0.5–1 kg exercises)
– 50–150 × 10^9^/L—progressive resisted exercises
– >150 × 10^9^/L—no restrictions.

### Assessment

Following the transplant and when medically appropriate, the individual is reassessed in line with the WHO ICF (37) by an appropriate MDT. Any potential areas of risk should be communicated to the specialist transplant therapy team before discharge. Any deterioration must be identified. If a patient's swallow has worsened, a bedside videofluoroscopy may be required.

### Advice and Information for Self-Management

Following the assessment, carers including nursing staff and family should be provided with manual handling advice to facilitate safe transfers and mobility whilst encouraging independence, provided there is no negative impact. They should also be provided with any positioning or seating guidance to optimize respiratory function and posture and to aid spasticity management if this continues to be a problem. These patients will also need advice on minimizing the risk of infections, which may include breathing exercises and be on an appropriate diet.

### Rehabilitation

The following should be assessed by an MDT as appropriate:

- Posture—in lying, sitting, standing, walking
- Seating
- Provision of any specialist equipment required
- Respiratory status
- Transfers and mobility
- Endurance
- Swallow
- Communication
- Cognition
- Personal activities of daily living and participation.

For specific symptom management, please see Multiple Sclerosis in Adults: Management (35). The following should be considered as part of rehabilitation:

– Cardiovascular exercise—many post-AHSCT patients have reduced aerobic capacity and decreased physical activity. Physical activity can restore exercise tolerance and improve function in patients after AHSCT (60).
– Progressive strengthening training can improve walking and balance but should be carefully prescribed for individual patients (53)
– Stretching programme as appropriate (55)
– Positioning and seating
– Spasticity management—commenced or continued with specific exercises, medication, and splinting as appropriate (35)
– Handling, facilitation, and specific exercise techniques for postural realignment
– Mobility progression (35)
– Balance exercises (35)
– Functional task practice
– Swallow guidance (35)
– Communication strategies (35)
– Cognitive rehabilitation (35, 57, 58)
– Fatigue management—please note that graded physical therapy has been shown to be effective in minimizing post-HSCT fatigue (61)
– Pain management (35).

### Intensity

During this phase, the patients need to be seen every day by a physiotherapist to ensure that their respiratory function is monitored and optimized and their physical function is assessed as well as receiving rehabilitation specific to their needs. Each patient will respond and react differently to the transplant process so each rehabilitation package is bespoke. Regular occupational therapy input is also required to ensure that independent function is optimized and speech and language therapy is provided as required.

## Rehabilitation During Phase 3: Sub-Acute Rehabilitation

Following the transplant and when medically stable, the individual should receive a period of intense inpatient or outpatient rehabilitation to optimize physical fitness, independence, and the outcome of the transplant. The timing of this will depend on the patient. This will likely be 8 weeks following the transplant, as prior to this, patients will be too weak to fully participate and are at the highest risk of EBV/CMV reactivation. Even with neutrophil recovery, the immune system remains suppressed for several months and patients may need readmission for infections and other complications. Therapists and patients also need to be aware that there is a risk of “late effects” associated with AHSCT. These may be the result of the transplant regimen and altered post-transplant immune reconstitution, but may also be driven by pre-treatment of the underlying neurological disease (19).

The rehabilitation in this phase should be aimed at treating both the neurological presentation and addressing the neuromuscular weakness and other disabilities now that the active inflammation has subsided.

Rehabilitation should include a full assessment and a treatment programme as above and should aim to further progress the patient. Therapy should consider cardiovascular workout equipment as well as the use of neuro-technologies where possible to optimize the effects of the AHSCT. Neuro-technology enables repetitive task practice. More evidence is emerging with regards to their benefits in neuro-rehabilitation (56). Neuro-technology can include the use of robotic gait training, upper limb devices, strength training, and balance devices as well as functional electrical stimulation and virtual rehabilitation (56). Care should always be taken to tailor these interventions to the individual patient and to monitor the impact of these technologies as our understanding of the long term impacts remain unclear.

Overall rehabilitation should follow a biopsychosocial, multi-disciplinary model with the patient having an allocated key worker, accessing the health care professionals required with a structured rehabilitation approach including access to groups and rehabilitation assistance sessions where appropriate. Utilizing a patient centered approach, patients and their carers should be involved with their goal setting. There should also be family meetings if required and opportunities for education to fully engage the patient in their rehabilitation. Following a self-management and self-care approach will ensure motivation, self-efficacy and the sustainability of behavior change once the patient has been discharged from formal rehabilitation.

### Intensity

The rehabilitation programme provided in this phase should be intensive. The intensity will vary depending on the patient and their pre-morbid and post-transplant presentation. For active individuals who tolerated the transplant procedure well, up to 4 h of therapy intervention could be received in a typical working day with input from physiotherapy, occupational therapy, speech and language therapy, and psychology as appropriate. Therapy groups can be considered providing immune status is taken into account.

### Advice and Information

Manual handling advice as well as seating and positioning programmes should be reviewed and any changes communicated as appropriate. As well as their therapy sessions, patients should be provided with exercise programmes to complete in their own time as part of their self-management programme. Patients should also be provided with advice regarding rest periods and fatigue management. The discharge planning and links into the community are made at this time. This includes exploring any on-going community support and return to work programmes.

## Rehabilitation During Phase 4: Community Rehabilitation Including Vocational Rehabilitation

This phase of rehabilitation occurs when the individual has been discharged home. Therapists and individuals need to be aware of late-effects of the AHSCT. A fever episode might cause deterioration in function but patients should be made aware that such conditions are usually fully reversible. The focus of rehabilitation during this phase is a continuum of the inpatient goals within their normal home environment, integrating the patient back into their home life and promoting independence. It is important that patients continue with their individualized rehabilitation programmes and progress their mobility and independence as able.

Returning to work in a timely manner is important for this patient group as many are in employment and may have dependants. Patients are generally not encouraged to return to work until 3 months post-transplant due to infection risks and in some cases delayed up until 6 months post-transplant depending on immune recovery. When and as appropriate, patients should be referred to appropriate vocational rehabilitation services for support. Specialist occupational therapists are crucial to carry out pre-employment physical and functional testing and liaising with the patient and their employer about returning to work (62). A specific return to work programme should be designed for the patient and progression monitored. Education of the employer is important as is linking into occupational health departments (62).

### Advice and Information

It is important that on-going support and guidance is available after the patient has returned home. This may require referrals to appropriate community-based teams including therapy teams, specialists (e.g., continence, sexual function, tissue viability) or social services. If appropriate, carers and family members should also be provided with advice and education to assist the individual going home. This may include advice on manual handling including the use of equipment, positioning, seating, and nutritional or feeding advice.

## Comprehensive Review

Regaining neurological function can continue in the first couple of years following AHSCT and it is important to optimize this period of time with a tailored, comprehensive rehabilitation programme. Patients who have undergone AHSCT should have a comprehensive review of all aspects of their care at least once a year, which needs to be carried out by healthcare professionals with expertise in MS and its complications. The review process and follow up treatments must be individually tailored to need. It may result in onward referral for further specialist support e.g., spasticity management, orthotics or specialist equipment available as required. Hydrotherapy can be considered providing immune status is taken into account by transplant physician and specialist hydrotherapist.

## Conclusions

The consensus recommendations are summarized in Table 1. Rehabilitation should be considered at each stage of the pathway on an individualized basis via a proper MDT assessment, with the aim of preventing secondary problems, reducing length of hospital stay and maximizing recovery and long-term outcomes. There is also potential to reduce long-term care costs. The lack of evidence-base to support the benefits (and risks) of rehabilitation in MS patients undergoing AHSCT is highlighted and further research is warranted. Table 2 summarises the future health research questions. The proposed framework for rehabilitation can be factored into clinical trials and future practice can be refined to reflect emerging evidence.

**Table 1:** Recommendations (all consensus).

• All patients with MS undergoing AHSCT should be considered for pre-habilitation before and rehabilitation following the procedure. Referrals should be made early so rehabilitation can be delivered at the optimal time. • Care should be provided with a co-ordinated multidisciplinary team (MDT) approach with a core and an extended team, who facilitate referral, assessment, and delivery of rehabilitation throughout the four phases of the pathway. • Rehabilitation should be goal directed and patient centered and underpinned by principles of self-management and self-care. • Rehabilitation for patients with AHSCT should be delivered at 4 phases during the pathway as detailed. • All individuals receiving AHSCT should be provided with appropriate advice and support about the process and the rehabilitation available to them. Advice leaflets are recommended to guide on exercise, nutrition, and any necessary lifestyle changes. If appropriate, carers and family members should also be provided with advice and education to assist the individual. Specialist consultation may be necessary in some cases. • Prior to the transplant, baseline level of impairment, function and participation should be ascertained through a formal MDT assessment process. Risk factors for potential deterioration should be identified. Following the assessment, carers including nursing staff and family should be provided with manual handling advice to facilitate safe transfers and mobility whilst encouraging independence. • A rehabilitation plan should be tailored to patients' symptoms and goals. Rehabilitation provided at each stage of the pathway will be dependent upon the clinical presentation. A series of validated outcome measures will be taken at key points throughout the patient's journey to monitor any change. • Validated and reliable standardized assessment tools and outcome measures should be recorded routinely to monitor any change. • Infection control measures and other precautions appropriate to the stage of AHSCT (such as thrombocytopenia) should be followed. • All MS patients who have received AHSCT should have a comprehensive review of all aspects of their care at least once a year, carried out by healthcare professionals with expertise in MS working as part of the extended MDT. Therapists need to be aware of “late-effects” of AHSCT as well as the potential for relapse and progression of MS.

**Table 2:** Future health research questions (consensus).

• Will rehabilitation in the AHSCT pathway result in further neurological improvement and long-term outcome? • Should a rehabilitation specialist be routinely part of the MDT to coordinate the rehabilitation and ensure outcome measures are obtained throughout the AHSCT pathway? • What are the best outcome measures to use in patients with MS undergoing AHSCT? Can specific outcome measures be developed for use with MS patients receiving AHSCT? • When is the optimal time to start rehabilitation in patients undergoing AHSCT? Does pre-habilitation have a role in AHSCT for patients with MS? • Should rehabilitation be provided to patients whilst in isolation, when they are most heavily immunosuppressed? If so, to what intensity? • Can length of hospital stay post-transplant be reduced? • Following discharge, should patients be referred into an intense sub-acute rehabilitation pathway either as an outpatient or into a step-down residential rehabilitation setting?

## Author Contributions

FR, HH, JS, BS, and HJ conceived the proposal and developed and wrote the manuscript with the other co-authors after approval by the EBMT Autoimmune Diseases Working Party (ADWP, Chair JS). FR, HH, CB, and SM are co-opted into the ADWP as physiotherapists with established experience and interaction with EBMT transplant centers and their patients. BS, JB, GM, PM, and AI are neurologists specialized in MS, including experience in AHSCT. JS, RS, MK, RG, KK, BW, and MP are hematologists experienced in AHSCT for MS. HJ is the EBMT Nurses Group representative on the ADWP. BV is the Chair of the EBMT Patient Advocacy Group, and thereby provided patient representation. FR and AI created the figures. All authors reviewed the final manuscript.

## Conflict of Interest

FR and HH work for Hobbs Rehabilitation, JS declares honoraria for speaking at educational meetings from Sanofi, Jazz, Janssen, Gilead, and Mallinckrodt, BV declares honoraria for speaking at educational meetings and consultancy from Janssen, Takeda, and Amgen. PM reports travel support and speaker honoraria from unrestricted educational activities organized by Novartis, Bayer HealthCare, Bayer Pharma, Biogen Idec, Merck-Serono, and Sanofi Aventis. He also discloses consulting to Magenta Therapeutics and Jasper Therapeutics. The remaining authors declare that the research was conducted in the absence of any commercial or financial relationships that could be construed as a potential conflict of interest. The reviewer EP declared a shared affiliation, with no collaboration, with two of the authors RS and CB to the handling Editor. The Handling Editor declared a past co-authorship with one of the authors GM.

## Abbreviations

AD: Autoimmune Disease
ADL: Activities of Daily Living
ADWP: Autoimmune Disease Working Party
AHSCT: Autologous Haematopoietic Stem Cell Transplantation
MDT: Multi-Disciplinary Team
MS: Multiple Sclerosis
OMs: Outcome Measures.
